# Disease-specific outcomes of Radical Prostatectomies in Northern Norway; a case for the impact of perineural infiltration and postoperative PSA-doubling time

**DOI:** 10.1186/1471-2490-14-49

**Published:** 2014-06-14

**Authors:** Sigve Andersen, Elin Richardsen, Yngve Nordby, Nora Ness, Øystein Størkersen, Khalid Al-Shibli, Tom Donnem, Helena Bertilsson, Lill-Tove Busund, Anders Angelsen, Roy M Bremnes

**Affiliations:** 1 Institute of Clinical Medicine, The Arctic University of Norway, Tromso, Norway; 2 Department Oncology, University Hospital of North Norway, Tromso 9038, Norway; 3 Institute of Medical Biology, The Arctic University of Norway, Tromso, Norway; 4 Department Pathology, University Hospital of North Norway, Tromso, Norway; 5 Department of Urology, University Hospital of North Norway, Tromso, Norway; 6 Department Pathology, St. Olavs Hospital, Trondheim University Hospital, Trondheim, Norway; 7 Department Pathology, Nordland Hospital, Bodoe, Norway; 8 Department of Urology, St. Olavs Hospital, Trondheim University Hospital, Trondheim, Norway; 9 Institute of Cancer research and Molecular Medicine, Norwegian University of Science and Technology, Trondheim, Norway

## Abstract

**Background:**

Prostate cancer is the most common male malignancy and a mayor cause of mortality in the western world. The impact of clinicopathological variables on disease related outcomes have mainly been reported from a few large US series, most of them not reporting on perineural infiltration. We therefore wanted to investigate relevant cancer outcomes in patients undergoing radical prostatectomy in two Norwegian health regions with an emphasis on the impact of perineural infiltration (PNI) and prostate specific antigen- doubling time (PSA-DT).

**Methods:**

We conducted a retrospective analysis of 535 prostatectomy patients at three hospitals between 1995 and 2005 estimating biochemical failure- (BFFS), clinical failure- (CFFS) and prostate cancer death-free survival (PCDFS) with the Kaplan-Meier method. We investigated clinicopathological factors influencing risk of events using cox proportional hazard regression.

**Results:**

After a median follow-up of 89 months, 170 patients (32%) experienced biochemical failure (BF), 36 (7%) experienced clinical failure and 15 (3%) had died of prostate cancer. pT-Stage (p = 0.001), preoperative PSA (p = 0.047), Gleason Score (p = 0.032), non-apical positive surgical margins (PSM) (p = 0.003) and apical PSM (p = 0.031) were all independently associated to BFFS. Gleason score (p = 0.019), PNI (p = 0.012) and non-apical PSM (p = 0.002) were all independently associated to CFFS while only PNI (P = 0.047) and subgroups of Gleason score were independently associated to PCDFS. After BF, patients with a shorter PSA-DT had independent and significant worse event-free survivals than patients with PSA-DT > 15 months (PSA-DT = 3-9 months, CFFS HR = 6.44, p < 0.001, PCDFS HR = 13.7, p = 0.020; PSA-DT < 3 months, CFFS HR = 11.2, p < 0.001, PCDFS HR = 27.5, p = 0.006).

**Conclusions:**

After prostatectomy, CFFS and PCDFS are variable, but both are strongly associated to Gleason score and PNI. In patients with BF, PSA-DT was most strongly associated to CF and PCD. Our study adds weight to the importance of PSA-DT and re-launches PNI as a strong prognosticator for clinically relevant endpoints.

## Background

Prostate cancer (PC) is the most common male malignancy and the second most common cause of cancer mortality in Norway [1]. It is presently the most prevalent cancer and there has been an increasing incidence until 2007 where after a reduction was noted [1]. When PSA was widely introduced in Norway in the early 1990s, an increasing number of men were diagnosed and treated with curative intent (33% in 2001) [2]. Although declining, Norway has the highest mortality rate of all western countries [3,4]. There has been an increasing use of radical prostatectomy (RP), but a majority of patients diagnosed with PC will not have symptomatic disease or die of the disease as they have non-lethal P [5]. The reduced risk of prostate cancer specific mortality after a radical prostatectomy compared to watchful waiting has been estimated to range between 0-25% depending on tumor characteristics [6]. The only RCT evidence for a reduction in prostate cancer mortality is the SPCG-4 trial [7].

Disease-specific survival after RP has mainly been reported from a number of single institutions, but a few studies have reported on larger cohorts and three studies have described survival in nationwide cohorts in Europe [8-10]. Some of these larger studies lack sufficient follow-up, relevant prognostic parameters and clinically relevant end-points in the analysis.

Clinicopathological variables for predicting disease outcome after RP are numerous with Gleason grade and score, pTNM-stage, preoperative PSA and surgical margins as the most widely adopted ones [11,12]. User-friendly predictive tools like look-up tables, risk classifications and nomograms have been developed [11,13]. Some variables like lymphovascular invasion [14], tumor volume [15], pT2 subclassification [16] and tertiary Gleason grade [17] are conflicting or have insufficient supporting data yet.

For patients with biochemical failure (BF) there are several treatment options including continuous, intermittent or deferred androgen deprivation, salvage irradiation of the prostate bed and trial participation. To choose the optimal therapy for these patients it is crucial to understand the risk factors for a subsequent clinical failure or death of PC, especially since management strategy remains controversial [18-20]. After BF, PSA kinetics, or more specifically PSA-Doubling Time (PSA-DT), has emerged as a prognostic variable [21-25]. However, as a pretreatment marker, PSA-DT has not found its position [26,27].

The objective of the present study was to describe disease outcome data for patients operated in the PSA era in three urological centers in two major health regions in Norway, and to examine the impact of post-prostatectomy PSA-DT on clinical outcomes.

## Methods

### Patients

671 patients were retrospectively identified with RPs for adenocarcinoma of the prostate between 01.01.1995 to 31.12.2005 from the archives of the Departments of Pathology at St. Olav Hospital/Trondheim University Hospital (St. Olav) (n = 341), Nordlandssykehuset Bodo (NLSH) (n = 63) and the University Hospital of Northern Norway (UNN) (n = 267). Of these, 131 patients were excluded due to non-available tissue blocks for re-evaluation (St. Olav n = 112, NLSH n = 3, UNN n = 15), four patients were excluded due to other cancers (not superficial skin cancers) within 5 years of diagnosis (UNN n = 4), one patient was excluded due to previous radiotherapy to the pelvic region (NLSH) and one patient due to lack of follow-up data (St. Olav). Thus, 535 eligible patients had complete follow-up data and tissue blocks for re-evaluation. Preoperative clinical TNM staging was unevenly stated in the medical files and data are therefore not presented.

### Definition of end-points and clinical variables

The preoperative PSA values were assessed right before surgery, except for those few patients who underwent transurethral resection of the prostate (TUR-P) prior to the RP. For these patients the PSA value before the TUR-P was used. PC was an incidental finding in these patients.

BF was defined as PSA ≥0.4 ng/ml in at least two consecutive postoperative blood samples according to Stephenson et al. [28]. Clinical failure (CF) was defined as verified symptomatic locally advanced progression after radical treatments and/or metastasis to bone, visceral organs or lymph nodes on CT, MR, bone scan or ultrasonography. Prostate cancer death (PCD) was defined as death with progressive and disseminated castration-resistant PC despite therapy. PSA-DT was calculated by the online available MSKCC-calculator (http://nomograms.mskcc.org/Prostate/PsaDoublingTime.aspx) which calculates a regression slope on the basis of all PSA values taken using natural log of 2 (0.693) divided by the slope of the relationship between the log of PSA and time of PSA measurement for each patient in months [24]. Up to four separate (at least 6 weeks apart) PSA measurements before supplementary treatment (endocrine therapy, radiotherapy or chemotherapy) were included. Optimal cut-off points for stratification of PSA-DT have been varying between reporters. We used cut-off values as the largest reported patient series to date from Johns Hopkins [21]. Hence, the four groups of patients with significantly differing prognosis for both CF and PCD were patients with PSA-DT <3 months, 3–9 months, 9–15 months and >15 months.

Postoperative follow-up (FU) protocols were not completely uniform in the participating hospitals, but all FUs included PSA measurements and clinical examinations (including digital rectal examination in PSA recurrence) every three months for the first year, every six months for the second year and once yearly for the following years. Imaging with CT, MRI or radio nucleotide bone scans was done upon symptoms or rising PSA.

The follow-up of patients was done by examining the patient medical files at the operating centers and the patients’ local hospital. Biochemical failure free survival (BFFS) was calculated from the date of surgery to the last FU date for BF, which was the last date of a measured PSA. Clinical failure free survival (CFFS) was calculated from the date of surgery to the last FU date for CF, which was the last date without symptoms or any evidence of metastasis. Prostate cancer death free survival (PCDFS) was calculated from the date of surgery to the date of death.

### Tissues

All prostate specimens were re-evaluated regarding to histopathological variables and re-staged according to the 2010 revision of the TNM classification [29] independently by two experienced pathologists (E.R, L.T.B). A positive surgical margin (PSM) was defined as tumor extension to the inked surface of the resected specimen [30-32]. Tumor size was measured as the largest diameter of the index tumor and was used due to previous observations of excellent correlation to PC volume [15]. Median tumor size was 20 mm and was set as cut-off in further analyses. Perineural infiltration (PNI) was defined as tumor cells within the perineural space adjacent to a nerve *outside* of the prostate capsule. Lymphovascular infiltration (LVI) was defined as tumor cells found within lymphatic or blood vessels. Gleason grading was re-graded according to the 2005 International Society of Urological Pathology Modified Gleason System [33].

### Statistical methods

Analyses for the patients with BF regarding the impact of PSA-DT on CFFS and PCDFS required the baseline date to be changed to date of BF as opposed to date of surgery for the other analyses.

The SPSS version 20 was used for the statistical analyses (Chicago, IL. USA). The non-parametric Spearman correlation coefficient (r) was used to calculate correlations between variables and only moderate or strong correlations (r > 0.3) are described. χ^2^ statistics were utilized for distribution differences between groups. Plots of the event-free survivals were drawn using the Kaplan-Meier method, and the statistical significance between survival curves was assessed by the log-rank test. Univariate analyses for the various endpoints (Table 1) according to clinical and histopathological variables were done. Significant variables (bold text in Table 1) were entered in the multivariate analyses for all patients (Table 2). The backward Cox regression analysis was used with a probability for stepwise entry and removal at 0.05 and 0.10, respectively. A p-value < 0.05 was considered statistically significant for all analyses. For the patients with BF (Table 3), all significant variables from the univariate analyses for both CF and PCD, were entered in the multivariate analysis These were tumor size, the margin variables, PNI, LVI, pT stage, pN stage and Gleason score. Due to the low number of events for PCD (15 events) we used an enter model with manual inclusion and removal of variables to identify the three most significant variables (Gleason score, PNI and positive non-apical margin) before entering them into the models.

**Table 1:** Patient characteristics and clinicopathological variables, and their prognostic value for the three endpoints in 535 prostate cancer patients (univariate analyses; log rank test)

**Characteristic**	**Patients**	**Patients**	**BF**		**CF**		**PCD**	
	**(n)**	**(%)**	**(170 events)**		**(36 events)**		**(15 events)**	
			**5-year EFS (%)**	**p**	**10-year EFS (%)**	**p**	**10-year EFS (%)**	**p**
**Age**				0.55		0.085		0.600
≤ 65 years	357	67	76		92		97	
> 65 years	178	33	70		88		96	
**pT-stage**				**<0.001**		**<0.001**		**0.027**
pT2	374	70	83		96		98	
pT3a	114	21	60		86		98	
pT3b	47	9	43		73		89	
**pN-stage**				**<0.001**		**<0.001**		**<0.001**
NX	264	49	79		95		98	
N0	268	50	71		89		97	
N1	3	1	0		33		67	
**Preop PSA**				**<0.001**		0.085		0.061
PSA < 10	308	57	80		93		99	
PSA > 10	221	42	67		88		95	
Missing	6	1	-		-		-	
**Gleason**				**<0.001**		**<0.001**		**0.001**
3 + 3	183	34	83		98		99	
3 + 4	220	41	76		93		98	
4 + 3	80	15	69		84		95	
4 + 4	19	4	63		76		94	
≥9	33	6	34		67		87	
**Tumor size**				**<0.001**		**0.019**		0.098
0-20 mm	250	47	82		94		99	
>20 mm	285	53	67		88		96	
**PNI**				**<0.001**		**<0.001**		**0.002**
No	401	75	79		95		98	
Yes	134	25	60		81		93	
**PSM**				**0.041**		**0.038**		0.697
No	249	47	81		94		97	
Yes	286	53	69		89		97	
**Non-apical PSM**				**<0.001**		**<0.001**		**0.029**
No	381	71	81		95		98	
Yes	154	29	57		81		94	
**Apical PSM**				**0.04**		0.484		0.31
No	325	61	73		90		96	
Yes	210	39	77		92		98	
**LVI**				**<0.001**		**<0.001**		**0.009**
No	492	92	77		93		98	
Yes	43	8	46		71		88	
**Surgical proc**				0.23		0.41		0.581
Retropubic	435	81	76		90		97	
Perineal	100	19	67		95		98	

*Abbreviations*: *BF* biochemical failure, *CF* Clinical failure, *EFS* event free survival in months, *LVI* lymphovascular infiltration, *PCD* prostate cancer death, *NR* not reached, *P* P value for log rank statistic for difference in event free survival, *PC* Prostate cancer, *PNI* Perineural infiltration, *Post op RT* postoperative radiotherapy, *Preop* preoperative, *PSA* Prostate specific antigen, *PSM* Positive surgical margin, *Surgical proc* surgical procedure. Significant p-values in bold (threshold p ≤ 0.05).

**Table 2:** Multivariate analyses in models including significant univariate analyses for all patients (Cox regression, backward conditional)

**Characteristic**	**BF (170 events)†**			**CF (36 events)**			PCD (15 events)*		
	**HR**	**CI95%**	**p**	**HR**	**CI95%**	**p**	**HR**	**CI95%**	**p**
**pT-stage**			**0.001**	NS			NS		
pT2	1								
pT3a	1.70	1.14-2.54	**0.010**						
pT3b	2.40	1.45-3.97	**0.001**						
**Preop PSA**				NE			NS		
PSA < 10	1								
PSA > 10	1.39	1.01-1.91	**0.047**						
**Gleason**			**0.032**			**0.019**			0.087
3 + 3	1			1			1		
3 + 4	1.05	0.70-1.56	0.81	2.45	0.78-6.90	0.09	3.71	0.41-33.2	0.242
4 + 3	1.55	0.97-2.47	0.07	2.87	0.91-9.10	0.07	10.47	1.21-90.7	**0.033**
4 + 4	1.42	0.68-2.97	0.36	2.73	0.52-14.2	0.23	7.43	0.46-121	0.159
≥9	2.39	1.31-4.35	**0.004**	6.74	2.21-20.6	**0.001**	15.26	1.65-141	**0.016**
**PNI**			0.090			**0.012**			**0.047**
No	1			1			1		
Yes	1.35	0.95-1.92		2.48	1.23-5.04		3.17	1.02-9.87	
**Non-apical PSM±**			**0.003**			**0.002**	NS		
No	1			1					
Yes	1.70	1.20-2.40		3.22	1.56-6.64				
**Apical PSM±**			**0.031**	NE			NS		
No	1								
Yes	0.69	0.49-0.97							

Significant p-values in bold (threshold p ≤ 0.05).

*Abbreviations*: *BF* biochemical failure, *CF* Clinical failure, *LVI* lymphovascular infiltration, *NE* not entered, due to non-significance in the univariate analyses, *NS* not significant, the characteristic is removed by the backward conditional analysis due to insignificance, *PCD* prostate cancer death, *PNI* Perineural infiltration, *Post op RT* postoperative radiotherapy, *Preop* preoperative, *PSA* Prostate specific antigen, *PSM* Positive surgical margin.

^†^Tumor Size, pN-stage and LVI were removed by the backward conditional model due to insignificance in all models.

^±^Only the subgroups (apical/non-apical PSM) of PSM were entered.

*Due to the low number of events the model was carefully analyzed in advance with the inclusion and removal of variables in an enter analysis to find the most significant in advance before doing the final model with the three variables; Gleason score, perineural infiltration and positive non-apical margin.

**Table 3:** Multivariate analysis including significant univariate analyses for the 170 patients with biochemical failure (Cox regression, backward conditional)

	**Patients**		**CF (36 events)†**					PCD (15 events)*				
	**N**	**(%)**	**5-year EFS (%)**	**HR**	**CI95%**	**p**	**Events (N)**	**10-year EFS (%)**	**HR**	**CI95%**	**p**	**Events (N)**
**PSA-DT**						**<0.001**					**0.029**	
Missing	12	7	-	NE			3	-	NE			0
>15	71	42	98	1	Ref		5	93	1	Ref		1
9-14.9	27	16	77	3.28	0.9-11.9	0.09	5	86	4.60	0.41-52.0	0.22	2
3-8,9	46	27	69	6.44	2.26-18.3	**<0.001**	16	70	13.7	1.51-124	**0.020**	8
<3	14	8	59	11.2	3.35-37.7	**<0.001**	7	49	27.5	2.64-286	**0.006**	4
**pN-stage**						**0.002**					**0.020**	
NX	69	41		1	Ref		9		1	Ref		3
N0	98	57		1.38	0.61-3.13	0.45	24		1.20	0.31-4.59	0.80	11
N1	3	2		19.1	3.69-100	**<0.001**	2		32.5	2.61-405	**0.007**	1

Significant p-values in bold (threshold p ≤ 0.05).

*Abbreviations*: *BF* biochemical failure, *CF* Clinical failure, *NS* not significant, the characteristic is removed by the backward conditional analysis due to insignificance, *NE* not entered, *PCD* prostate cancer death, *PSA* Prostate specific antigen, *PSA-DT* PSA doubling time in months; ^†^a positive non-apical margin, PNI, vasc inf, pT stage and Gleason were removed by the backward conditional model due to insignificance in all models. *Due to the low number of events the model was carefully analyzed in advance with the inclusion and removal of variables in an enter analysis to find the most significant in advance before doing the final model with the three variables; PSA-DT, Gleason score and pN-stage.

### Ethics

This study was approved by the regional ethics committee, REK Nord, project application 2009/1393.

## Results

### Patient characteristics

Median age at surgery was 62 years (range 45–75), median follow-up of survivors was 89 months (range 6.3-188.3). 279 patients underwent a limited lymphadenectomy.

The pT2 group (n = 374) was sub-classified to pT2a (n = 139; 37%), pT2b (n = 34; 9%) and pT2c (n = 201; 54%). pT stage was correlated to PNI (r = 0.33, p < 0.001), T-Size (r = 0.30, p < 0.001) and positive non-apical margin (r = 0.373 p < 0.001).

Indications for lymph node dissection were not predetermined for the centers involved, but was done according to the surgeons preference which mostly was if Partin nomograms indicated >10% risk of N1 disease or Gleason ≥8, PSA ≥10 or suspected cT3. Three patients were found to have discrete metastasis in regional lymph nodes at re-evaluation (paraffin embedded tissue) of initial frozen-section-negative lymph nodes.

Preoperative PSA was available for 542 of 548 (99%) patients. Median value was 8.8 ng/ml (range 0.7-104.3). The variable was dichotomized with PSA = 10 ng/ml as chosen cut-off.

Distributions of Gleason scores are presented in Table 1. Patients with Gleason 4 + 5 (n = 26; 5%), Gleason 5 + 4 (n = 6; 1%) and Gleason 5 + 5 (n = 3, <1%) were pooled in the Gleason ≥9 group due to the low number of these patients. In patients with BF, Gleason score correlated inversely with PSA-DT (r = -0.37, p <0.001)

The maximum diameter of the index tumor (T-Size) had a median value of 20 mm (range 2–50). T-Size correlated significantly with pT stage (r = 0.30 p < 0.001). We explored the prognostic value of T-Size in the pT2 subgroup, but no significant association to event-free-survival was found in univariate analyses and the variable was consequently not entered into multivariate analysis.

PNI correlated to LVI (r = 0.393, p < 0.001), and pT stage (r = 0.33, p < 0.001 77 (14%) patients of the patients had both apical and non-apical PSM. There was a significant decline of PSMs for RPs performed during the latter part of the period (χ2, p < 0.001). During the period 1995–2000 129/197 (66%) patients had PSM while in the period 2001–2005 162/351 (46%) patients had PSM. LVI correlated with PNI and to pT stage (r = 0.33, p < 0.001.

In pT3 patients, 109 of 161 (68%) patients had PSM while 182 of 387 (47%) pT2 patients had PSM. There was a significant association with operating center (χ2, p < 0.001) with St. Olav having the lowest PSM rates.

In non-apical PSM, PSA correlated significantly with pT stage (r = 0.373, p < 0.001).

When stratifying for pT stage non-apical PSM had a significant impact in pT3a patients (p = 0.001), but not in pT2 (p = 0.69) or pT3b patients (p = 0.075).

### Events and PSA-DT

**Figure 1:**
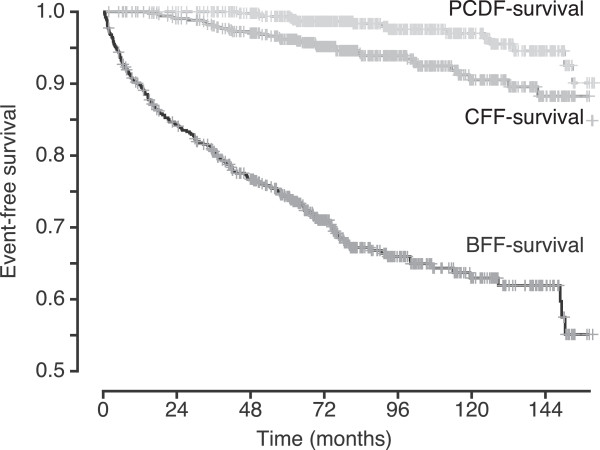
**Overlapping Kaplan-Meier curves, each illustrating event-free survival for the whole cohort for the specific event.** Note that the Y-axis has been modified with a proportion of 0.5 as the origin to better illustrate the differences. PCDF-survival = Prostate cancer death free-survival; CFF-survival = Clinical failure free-survival; BFF = Biochemical failure free-survival.

170 patients had BF during FU. Of these, 31 patients never reached postoperative PSA nadir < 0.4 ng/ml. When removing these patients (143 patients left) the median time to BF was 35 months (range 2.8-164). For CF patients the median time from BF to CF was 38.2 months (range 0–130.7). For PCD patients the median time from BF to PCD was 72.2 months (range 34.4-147). Kaplan-Meier curves illustrates event-free survivals are in Figure 1.

**Figure 2:**
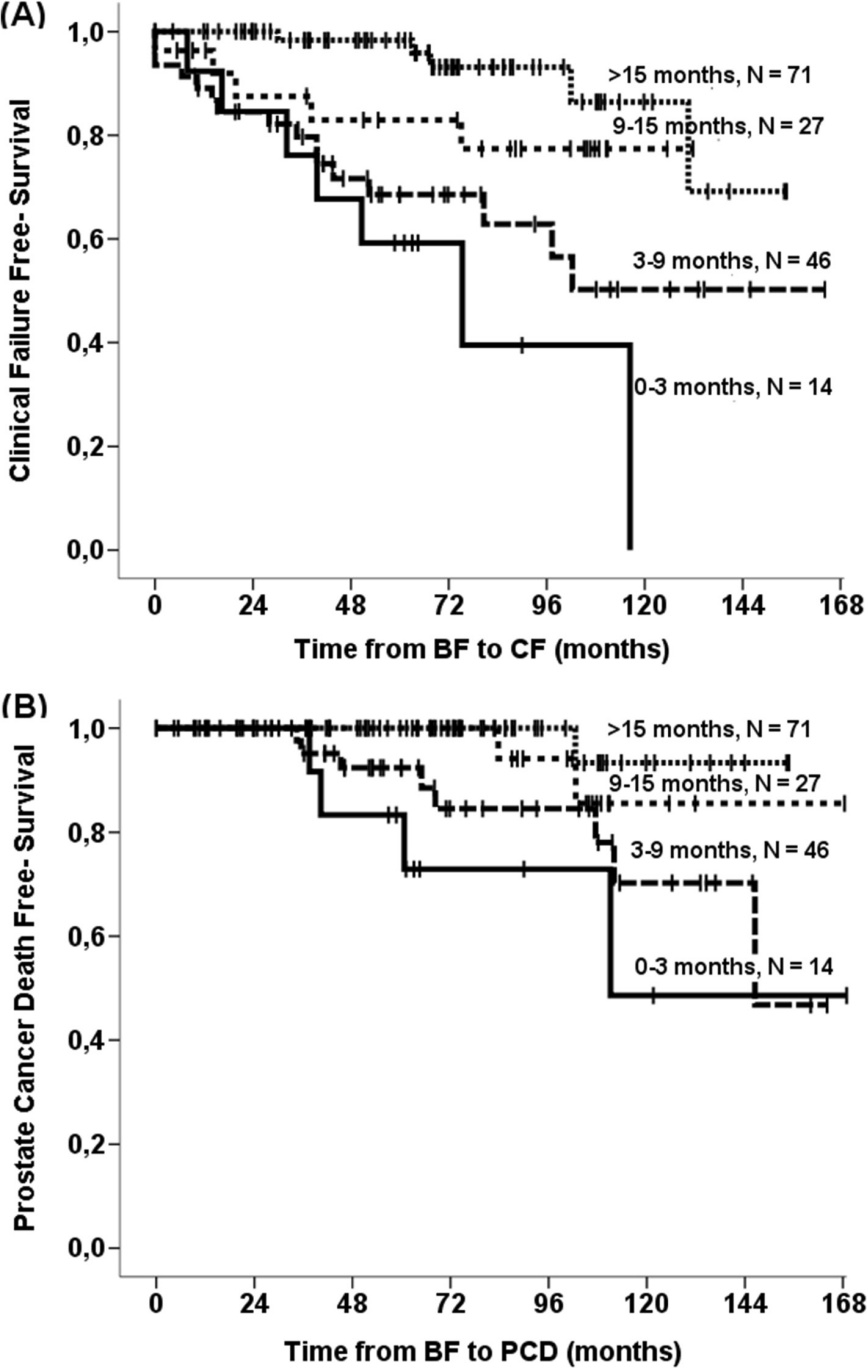
**Kaplan-Meier curves of patients experiencing biochemical failure for (A) Clinical failure free-survival stratified by PSA-doubling time categories and (B) Prostate cancer death free-survival stratified by PSA-doubling time.** See Table 3 for details regarding hazard levels and level of significance.

PSA data before salvage therapy was retrievable for 158 out of 170 patients with BF to calculate PSA-DT. Median PSA-DT was 13.6 months (range 0.4-332). Quartile cut-off values were 5.5, 13.4, and 23.9 months. We used the previously published cut-offs regarding PSA-DT [21] as these in our cohort reliably divided the patients into subgroups with differing hazard ratios for CF or PCD. PSA-DT correlated inversely with Gleason score (r = -0.37, p < 0.001) (Figure 2). Kaplan-Meier curves illustrating event-free survivals according to different PSA doubling times are in Figure 2.

For validation we did subgroup analyses stratifying for health regions. The results were highly significant in both Helse Nord (p < 0.001) and Helse Midt (p = 0.004).

Among the 36 (7%) patients with CF, 13 patients had symptomatic locally advanced progression, 18 had bone metastasis and 5 had regional lymph node metastasis. Median time to CF after RP was 64 months (range 15–159).

Concerning CF, 8/33 events (24%) were in patients with PSA-DT < 3 months and 23/33 events (70%) were in patients with PSA-DT less than 9 months. For PCD, 4/15 events were in patients with PSA-DT < 3 months and 12/15 events were in the patients with PSA-DT less than 9 months.

15 patients died of PC leaving 43/58 (74%) patients to have died of other causes at a mean time of 76 months after RP.

### Impact of surgical center

Patients had their RP at one of three hospitals: UNN (n = 248, St.Olav (n = 228) and NLSB (n = 59). There was no statistical significant differences in risk of CF (p = 0.40) and PCD (p = 0.973) between the centers.

## Discussion

This is the first large Scandinavian multicenter study presenting the impact of prognostic variable information regarding BF, CF and PCD in Scandinavia in the PSA era. We found post RP PSA-DT to be a strong predictor of CF and PCD, even outperforming Gleason score. In addition we found PNI to be an independently strong predictor of all event-free survivals. Otherwise we found the prognostic factors in this material to be mostly consistent to previously published larger series [34-36].

The strength of this paper is the unselected study population from Central and Northern Norway. It is reasonable to assess that more than 95% of men diagnosed with PC in this geographical area, were operated in hospitals that participated in this study. Moreover, the study has a relatively long FU, only patients in the PSA era were included, all tissues have been re-evaluated by two experienced pathologists, few patients had missing PSA-DT information, we have included relevant prognostic factors, and adjuvant treatment after RP was rare in the timeframe of this study. Weaknesses of the study are the retrospective design, the probable impact of salvage radiotherapy on the risk of time to events and events are low at longer follow-up times, contributing to low precision and large CIs (Tables 2 and 3). In addition, a number of tissue blocks were missing from one center, thereby reducing representativity.

Like other studies, we have found that the time from RP to CF and death of PC to be extensive, even in patients with a BF [21,37]. Prognostic factors to stratify patients for risk-adapted follow-up, treatment regimens or clinical trials are crucial since the majority of operated patients will not have BF. Furthermore an even greater majority will not experience symptoms of their disease and only a very few will die of PC. On the other hand, it will be important to identify those patients who otherwise will have symptoms from the recurrent disease or die. Another interesting observation is that the involved surgeons seem very capable to select patients for surgery with a long expected survival as only 8% patients died of other causes during follow up.

Our observation of post-prostatectomy PSA-DT as the strongest predictor of CF and PCD in patients with BF is consistent with numerous other studies recognizing the importance of PSA-DT as a predictor of CF [21,22,38] and PCD [37,39,40] after RP. We found the same pattern in both health regions, thereby internally validating these results. The importance of PSA-DT in patients treated with radiotherapy has also been reported [41]. In accordance with Antonarakis et al. we found patients in the two lowest PSA-DT categories (<3 months and 3–8,9 months) to have the worst prognosis with comparable Hazards ratios in the multivariable analysis. 64% of CF events and 80% of PCD deaths were in these two groups even though they collectively constituted only around 1/3 of the patients (35%). Although there was a correlation between Gleason Score and PSA-DT we saw the same trends of poor event-free survival when stratified for Gleason Score subgroups, but numbers were statistically insignificant due to the low number of patients in each subgroup. Our observation adds weight and validates the importance of PSA-DT for selecting BF-patients at high risk of developing clinically significant disease in the future.

At time of diagnosis, Gleason score has been shown to be a strong predictor of high risk PC [42], but also metastasis and PCD after RP [38,43]. Including all patients in the analyses we consistently found patients with a Gleason sum ≥9 to have the highest risk of BF (equal to pT3b), CF and PCD. This highlights a major impact of Gleason score in risk stratification following RP. Some have, however, suggested that Gleason score loses its value after including PSA-DT in the risk-stratification [44]. Analyzing patients with BF only, the PSA-DT removed Gleason score from the step-wise multivariate analyses due to its co-variation and prognostic strength. Antonarakis et al. found both Gleason score and PSA-DT to contribute to estimate metastasis free-survival [21] although PSA-DT was the strongest predictor.

Herein, extraprostatic PNI was in addition to Gleason score the only clinicopathological variable to predict both CF and PCD. Patients with an observed extraprostatic PNI had estimated HRs of CF and PCD at around 3. The ability of PNI to predict subsequent events has been controversial. Some have found PNI to independently predict BF [45-48] while others did not found PNI to be a predictive factor for any event [49-53]. D’Amico et al. reported PNI to be an independent predictor of BF, but only for low risk PC after evaluating biopsies [54]. Besides, the evaluation of its role as a prognostic factor has been hampered as it does not seem to be included as a histopathological variable in the large series of the world e.g. the Johns Hopkins database [55]. Most studies have only addressed the correlation between PNI and BF and not the more clinical relevant endpoints of CF and PCD. In the small, but interesting study by Aumayr et al. reported that a high amount of extraprostatic nerve infiltration correlated with tumor progression [45]. PNI found in preoperative biopsies, has also been found to be a predictor of metastasis and PCD in patients treated with dose-escalated radiotherapy [56]. Our finding of extraprostatic PNI as independently significant for prognosis with respect to CF and PCD, but not for BF, is in accordance with these findings.

PSM rates in our material are high (overall 53%) and among the highest that have seen published. In a large published single-center series from Mayo by Boorjian et al. in an almost identical period of time they found a PSM rate of 31.1% with an decreasing incidence over time [57]. The explanation could be the high incidence of pT3x cancers (30%) compared to 12% in the Mayo cohort and higher Gleason grades in our material, as these are independent predictors of PSM. In addition, as our centers during this timeframe were low-volume centers by international standards, this may have contributed to the high PSM rates [58]. Refinement of surgical technique and stage migration has been documented to improve the histopatological outcomes [59,60]. Margin location was relevant in our cohort, as non-apical PSM were associated with a poor BFFS and CFFS. Margin localization was not analyzed by Boorjian et al., but in a study from the same institution by Blute et al., they found PSM at the prostate base to be independently associated to outcome, as did Obek et al. [61,62]. A study by Godoy et al. found the base and anterior localization of PSM to be independently associated to an increased risk of BF compared to the other margin localizations. They specifically advocate that there is over-reporting of PSM from the apex and that an observation strategy is to be adopted for the large group of patients with a apical localization of PSM [63]. A Danish study also found non-apical PSM to be independently associated to BF, whereas apical margins were insignificantly associated to BF in multivariate analysis [64].

## Conclusions

In conclusion, for the minority of patients with a subsequent BF we found a low PSA-DT to be the strongest prognosticator for CF and PCD, recognizing its superiority in risk-stratification in this subgroup. We also re-launch PNI in the pathological specimen as a possible strong predictor of CF and PCD following RP and a thorough evaluation in larger patient series is warranted.

As most patients, even after risk-stratification, will not experience a clinically significant relapse of the disease we need new prognostic markers to identify the relevant subgroups. We have included tissues from tumor and stroma of these thoroughly described and largely unselected patients in tissue micro array blocks. Hence, this forms an excellent platform for future molecular studies which will hopefully give us some of these answers.

## Competing interests

The authors declare that they have no competing interests.

## Authors’ contributions

Collecting clinical data: SA, YN, NN, HB. Revising and collecting pathological data: ER, ØS, KA-S, L-TB. Drafting the manuscript: SA, ER, AA, RMB. Statistical analysis: SA, TD, YN. Design of study: SA, ER, TD, HB, L-TB, AA, RMB. All authors have read, revised and approved the final manuscript.

## Pre-publication history

The pre-publication history for this paper can be accessed here:

http://www.biomedcentral.com/1471-2490/14/49/prepub
